# “Digital self-harm” as a double-edged sword: a case study

**DOI:** 10.1192/j.eurpsy.2025.1129

**Published:** 2025-08-26

**Authors:** C. García Cerdán, M. Ligero Argudo, I. M. Peso Navarro, I. Rodríguez Blanco, C. Munaiz Cossio, B. Refoyo Matellán, P. Salas Aranda

**Affiliations:** 1 Psychiatry, University Clinical Hospital of Salamanca, SALAMANCA, Spain

## Abstract

**Introduction:**

Digital self-harm refers to the use of information and communication technologies (ICTs) to post or share self-deprecating or harmful content. This often occurs on forums or social networks, where the use of verbal and non-verbal codes (hashtags, emojis) complicates external monitoring. The rise in ICT usage and self-destructive behaviors online has raised concerns among mental health and education professionals, as these actions are linked to conditions such as depression, anxiety, and traditional physical self-harm in young populations.

**Objectives:**

To explore the phenomenon of digital self-harm and its effects on adolescents.

To examine the role of AI tools in addressing this issue through a clinical case.

**Methods:**

A brief literature review on digital self-harm.

Analysis of a clinical case: A 15-year-old female with a history of multiple hospitalizations due to suicidal risk and self-harming behaviors. Under treatment with Venlafaxine and Aripiprazole, she is diagnosed with “emotion dysregulation disorder of adolescence” and “mixed adaptive disorder.” Her social interactions are mostly limited to online networks. She describes her self-harm as “addictive,” used not only as an anxiolytic but as a way to achieve “social positioning,” posting about it in forums and comparing herself with others. When she reported her self-harm impulse to an AI (artificial intelligence) chatbot for suicide prevention, emergency services were activated, leading to her hospital admission.

**Results:**

International studies indicate that between 6% and 9% of adolescents have engaged in digital self-harm behaviors. In Spain, reports from the ANAR Foundation and UNICEF have shown an increase in this phenomenon since the Covid-19 pandemic. These “online support communities” can foster dynamics of rivalry and become harmful, as they not only share images but also techniques to avoid detection or hospitalization. In the presented case, the patient’s initial isolation led her to use ICTs as a way to seek social affirmation and a sense of belonging. Upon encountering specific forums, self-harm, which initially served an anxiolytic function, evolved into a mechanism for achieving social relevance. On the other hand, the AI chatbot for suicide prevention facilitated early intervention in her case.

**Conclusions:**

While the internet can provide social support for isolated adolescents, it also has the potential to normalize and even reinforce self-harming behaviors among vulnerable populations. Therefore, it is crucial to further investigate the psychosocial factors involved in digital self-harm and to develop new tools for mental health professionals. Additionally, AI could serve as an entry point and tool for younger generations, offering potential for both prevention and therapeutic intervention.

**Disclosure of Interest:**

None Declared

